# CAD systems for COVID-19 diagnosis and disease stage classification by segmentation of infected regions from CT images

**DOI:** 10.1186/s12859-022-04818-4

**Published:** 2022-07-06

**Authors:** Mohammad H. Alshayeji, Silpa ChandraBhasi Sindhu, Sa’ed Abed

**Affiliations:** 1 Computer Engineering Department, College of Engineering and Petroleum, Kuwait University, P.O. Box 5969, 13060 Safat, Kuwait City, Kuwait; 2 Different Media, P.O. Box 14390, Faiha, Kuwait

**Keywords:** Computer-aided diagnosis, COVID-19, Computed tomography, Deep neural network, Semantic segmentation, Machine learning, Severity score, Classification

## Abstract

**Background:**

Here propose a computer-aided diagnosis (CAD) system to differentiate COVID-19 (the coronavirus disease of 2019) patients from normal cases, as well as to perform infection region segmentation along with infection severity estimation using computed tomography (CT) images. The developed system facilitates timely administration of appropriate treatment by identifying the disease stage without reliance on medical professionals. So far, this developed model gives the most accurate, fully automatic COVID-19 real-time CAD framework.

**Results:**

The CT image dataset of COVID-19 and non-COVID-19 individuals were subjected to conventional ML stages to perform binary classification. In the feature extraction stage, SIFT, SURF, ORB image descriptors and bag of features technique were implemented for the appropriate differentiation of chest CT regions affected with COVID-19 from normal cases. This is the first work introducing this concept for COVID-19 diagnosis application. The preferred diverse database and selected features that are invariant to scale, rotation, distortion, noise etc. make this framework real-time applicable. Also, this fully automatic approach which is faster compared to existing models helps to incorporate it into CAD systems. The severity score was measured based on the infected regions along the lung field. Infected regions were segmented through a three-class semantic segmentation of the lung CT image. Using severity score, the disease stages were classified as mild if the lesion area covers less than 25% of the lung area; moderate if 25–50% and severe if greater than 50%. Our proposed model resulted in classification accuracy of 99.7% with a PNN classifier, along with area under the curve (AUC) of 0.9988, 99.6% sensitivity, 99.9% specificity and a misclassification rate of 0.0027. The developed infected region segmentation model gave 99.47% global accuracy, 94.04% mean accuracy, 0.8968 mean IoU (intersection over union), 0.9899 weighted IoU, and a mean Boundary F1 (BF) contour matching score of 0.9453, using Deepabv3+ with its weights initialized using ResNet-50.

**Conclusions:**

The developed CAD system model is able to perform fully automatic and accurate diagnosis of COVID-19 along with infected region extraction and disease stage identification. The ORB image descriptor with bag of features technique and PNN classifier achieved the superior classification performance.

## Background

The lung is a respiratory organ which is powerless against airborne injuries and contaminations. According to World Health Organization (WHO) [1] recent reports, third most common cause of death is lung diseases, with about three million people dying per year. Even though smoking, genetics, and air pollution are among the causes of lung diseases, the main current reason for the huge rise in lung disease is infection by bacteria or viruses. Acute lower respiratory tract infections appear to be a primary cause of death and illness in both children and adults. Bacteria or virus lung infections affect the lungs functionality and may even lead to death if not treated on time. SARS-CoV-2 (severe acute respiratory syndrome coronavirus 2) is a recent human pathogenic virus that causes severe lung disease and has impacted several million people worldwide [2]. This disease first reported in China’s Hubei Province in or before December 2019 and had spread internationally by early. The immune system will experience symptoms within two to fourteen days if the virus gets into touch with the mucous membranes that line a person's mouth, nose and eyes. By entering into healthy body cells, the virus kick-starts the production of more infected cells. Soon, it multiplies and infects the rest of the cells. Viral proteins use angiotensin-converting enzyme 2 (ACE2) receptors to gain entrance to healthy cells, where they then seize them. Having taken command of such cells, these proteins continue to destroy them completely. As there are more ACE2 receptors in the lower airways than in other places, COVID-19 is more likely to travel deeper into the respiratory tract. Human respiratory tracts are affected by the virus either at the upper or lower parts; then the immune system tries to fight the infection as it passes through the respiratory tract. Infection caused by the virus results in lung and airway swelling and inflammation. The infection usually starts in one part of the lung and may then spread further.

COVID-19 causes lung complications, such as pneumonia, resulting in shortness of breath caused by fluid build-up in the lungs. Furthermore, lung inflammation inhibits the ability to absorb oxygen. Based on the level of infection within the lungs, the disease can be mild, moderate, or severe. Because of the huge rise in patient counts on daily basis, hospitals are struggling to provide treatment that keeps up with hospital admission rates. Chest X-ray (CXR) and CT scans are the medical imaging tools employed to diagnose COVID-19. These imaging modalities are also highly helpful in the early diagnosis of lung diseases. Samples taken from the nose and throat are used to determine whether COVID-19 is present through real-time reverse transcription-polymerase chain reaction test (RT-PCR). Some studies, however, have already reported that it is less sensitive during the initial disease stages and that 24 h are required for a result to be confirmed. Physicians can identify a more detailed disease picture by using a CT scan than by using conventional X-rays. Moreover, a CT scan can identify the exact problem location more precisely [3]. The common CT findings of COVID-19 patients are ground-glass opacities (GGOs), peripheral distribution, multilobar involvement, bilateral lesion involvement, and posterior lesion topography [4]. Largely, however, GGOs are seen with crazy-paving patterns, and nodular or rounded features. The lower lobes of the lungs are the most affected by this pneumonia and, in most cases, GGO findings are visible even in the initial disease stages. Specifically, CT images give clearer and more detailed information about the lung region than CXR.

CT scan is selected as the imaging modality for the model development since it can identify the exact problem location more precisely. Although simple conventional segmentation methods exist, they are not effective for our purpose due to the database diversity. Recently, deep neural networks have attracted many researchers, who have used different deep learning (DL) models for semantic segmentation. Here performed two classes of segmentation (background and lungs) in the diagnosis stage for the ROI extraction, since we were able to achieve computational efficiency in the later stages. Once diagnosed with COVID-19, the infected regions were segmented by using a three-class segmentation (i.e., background, lungs, and COVID-19-infected regions). This semantic segmentation was achieved using the most recent, fastest and computationally efficient semantic segmentation network DeepLabv3+, which was invented by Google [5]. In the feature extraction stage, scale-invariant feature transform (SIFT), oriented FAST and rotated BRIEF (ORB) and speeded-up robust features (SURF) techniques [6] were implemented to ensure the model was invariant to scale, rotation, distortion, noise, etc. Due to the direct feature engineering involved in classical ML, these algorithms are quite easy to interpret and understand. Hence, we finalized with conventional ML classifiers for the proposed model development.

**Fig. 1:**
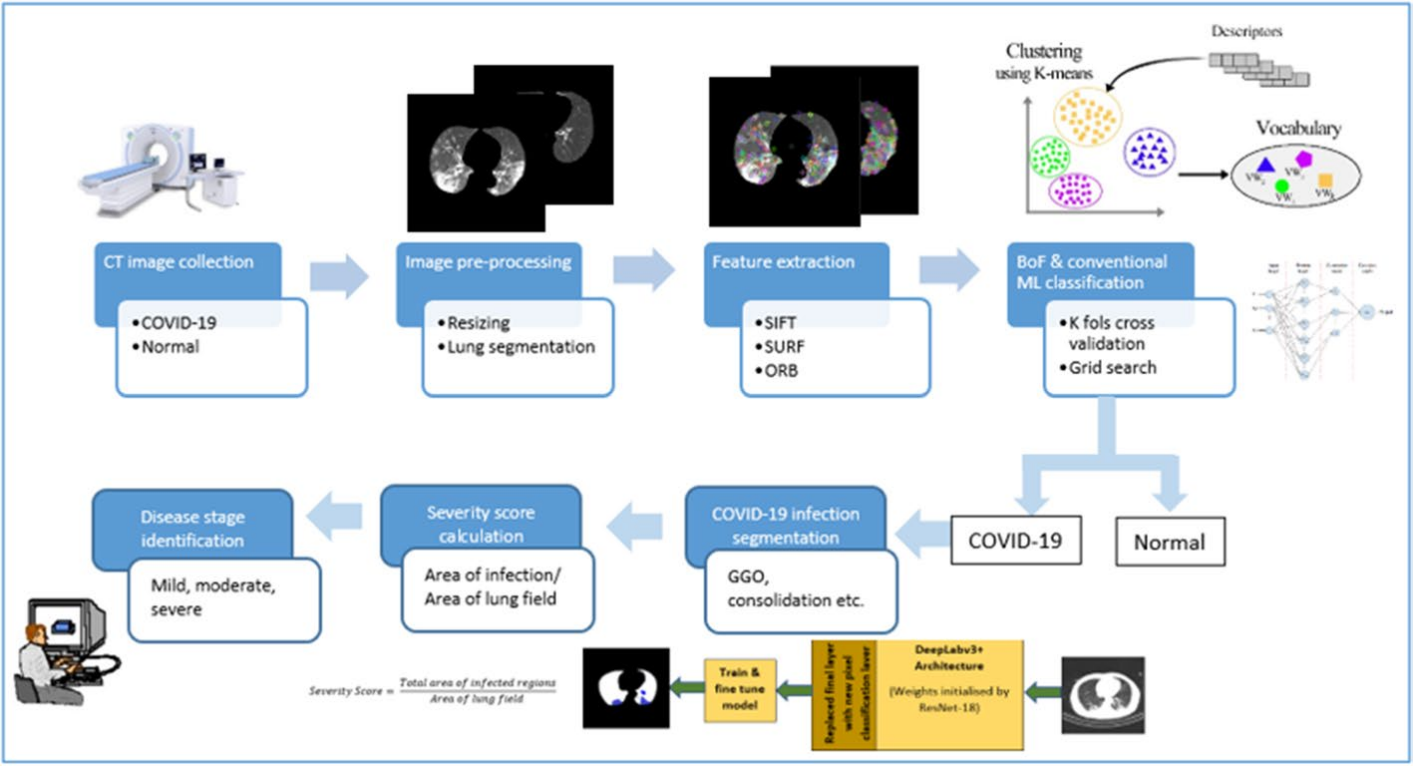
Workflow diagram

The complete workflow is illustrated in Fig. 1. The major contributions of this paper are outlined below.

1. Developed an automatic CAD system able to perform COVID-19 diagnosis by utilizing lung CT images with the help of conventional ML steps. Also, implemented bag of features technique followed by SIFT, SURF, and ORB image descriptors in the feature extraction stage of the CAD system. Applying these image descriptors helps to differentiate between COVID-19 affected and normal lung CT images accurately and training the model with this information helps to achieve high performance. To the best of our knowledge, this is the first work utilizing this feature extraction technique for COVID-19 diagnosis.
2. Developed a DL semantic segmentation method for the segmentation of infected regions, as identified via lung CT scans of COVID-19 patients and visualized it.
3. Carried out a severity score evaluation implementation on the developed CAD system, allowing for infection stages to be identified without the need for medical professionals and for appropriate medical assistance to be given at time of hospital admission without delay.
4. Used a diverse dataset with a huge number of CT images to achieve a real-time applicable model. Moreover, here experimented with different networks in the DL model and employed transfer learning, grid search (GS), and cross-validation concepts.

## Related works

From the starting stage of COVID-19 pandemic itself, researchers from different areas started working on diagnosis application to come up with useful findings that will aid in automatic diagnosis systems.

### DL classification approaches

Majority of the COVID-19 diagnosis research works were purely based on DL networks. Silva et al. [7] employed a high-quality DL model for COVID-19 diagnosis with EfficientNet by implementing a voting-based approach and cross dataset study, using the two largest publicly available datasets. The major limitations on the use of CT scan images were that slices from the same patients were treated independently, and images from the same patient could be repeated in the train and test dataset studies. In their study, this issue was solved by the concept of the voting-based approach. The voting scheme considered all CT images of a given patient rather than a single CT image; hence, it gave a high success rate. Jin et al. [8] developed and deployed a COVID-19 diagnosis system in four weeks, using a limited CT image dataset that was available in the COVID-19 pandemic initial stage. In this work, the authors performed 3D segmentation and classification as key stages using 3DUnet++-ResNet-50. Later, in the research by Santosh et al. [9], a type of active learning was used in which the learner had some role in deciding the data trained; hence, it was a kind of self-learning. This kind of incremental learning helps the model adapt to a new kind of dataset without losing knowledge of an existing one. Furthermore, an anomaly detection technique was employed to access the changes in data.

In [10], DL was utilized to train X-ray and CT-scan images individually. The upgraded VGG16 deep transfer learning models are used to perform COVID-19 classification. For COVID-19 CT-scan image binary classification, they employed four pre-trained convolutional neural network (CNN) models: VGG16, DenseNet121, ResNet50, and ResNet152, and suggested the fast AI ResNet framework in the detection of COVID-19 CT-scan images with high accuracy of 99%. However due to the limitation regarding the metadata, they were unable to incorporate disease severity identification module into their framework. A novel deep neural network architecture that is tailored for the detection of COVID-19 cases from CXR images using a human–machine collaborative design strategy named COVID-Net was implemented in [11]. When employing COVID-Net for accelerated computer-aided screening, COVID-Net produces predictions using an explainability method in an attempt to acquire deeper insights into crucial factors connected with COVID cases, which can benefit clinicians in enhanced screening as well as promote trust and transparency. This approach achieved 98.9% positive predictive value (PPV) but failed in predicting the risk status.

The primary goal of Kassania et al.’s [12] work was to implement a generic feature extraction method using a CNN to eliminate the handcrafted and complex features needed for imaging modalities as well as to reduce generalization error and increase diagnosis accuracy. In this study, they employed 15 different CNN feature extractors and 6 ML classifiers for COVID-19 identification from normal cases, using X-rays and CT scan images. Since they lacked sufficiently large training data to develop the model from scratch, they used a transfer learning concept which also eliminated the problem of overfitting. To achieve better generalization, they also avoided data augmentation and extensive pre-processing. Here authors state that avoiding extensive preprocessing helps to make the model more robust to noise, artifacts and variations in input images during feature extraction phase, and avoiding data augmentation will reduce bias toward the model performance. In this work, they concluded that combinations of deep CNN and bagging tree classifiers give better classification performances.

All these reviewed models completely relied on the DL networks in taking COVID-19 diagnosis decisions. Since they were acted like black boxes it is unable to identify the criteria based on which network took such decisions. In the DL approach applied by Gozes et al. [13], abnormalities were visualized using grad-CAM technique by extracting activation functions, since these contribute to the area responsible for a DL network’s decision. Similarly, grad-CAM visualization used in [14], where transfer learning implemented to test COVID-19 using CT images and analyzed the effects of various starting parameters on the results. They demonstrated that the model, which was pre-trained on ImageNet21k, have strong generalizability in CT images and the model achieved an accuracy of 99.2%.

### Classical ML approaches

Only few research work carried out with classical ML approach in COVID-19 diagnosis where hand crafted features come into action. Al-Karawi et al. [15] proposed an ML approach to find COVID-19 patients, using a texture analysis concept in the feature extraction stage by employing a fast Fourier transform (FFT) Gabor scheme. And achieved an average accuracy of 95.37%, along with very low false negatives. They were also able to visually give evidence by displaying the final features on which the prediction decision was based. In [16], Barstugan et al. used Grey-level co-occurrence matrix (GLCM), grey-level run length matrix (GLRLM), grey-level size zone matrix (GLSZM), local directional pattern (LDP) and discrete wavelet transform (DWT) algorithms as feature extraction methods. Abd Elaziz 2020 et al. [17] utilized orthogonal moment feature properties and feature selection techniques. Extraction of features were carried out by new fractional multichannel exponent moments (FrMEMs), and a new feature selection method was employed by improving manta ray foraging optimization (MRFO) using differential evolution (DE). Patel et al. [18] used features such as, clinical, blood-panel profile and socio-demographic data for severity identification and stated that the ML model with random forest (RF) gives the most accurate critical and mechanical ventilation prediction. The authors in [19] used clinical information along with CT images, including count of leukocyte, absolute lymphocyte number, neutrophils and lymphocytes percentage. In the classification stage, they used SVM, multilayer perceptron (MLP) and RF classifiers, of which the MLP performed well. Finally, the model was created by the combination of radiological and clinical information.

### Lung infection segmentation approaches

All the reviewed works lack separate COVID-19 infection region extraction after COVID-19 classification. This part is important to help clinicians for taking vital decisions in timely manner. In [20] they present CoSinGAN, a new conditional generative model that can be learned from a single radiological picture with a certain condition, such as the lungs and infected regions annotation mask. Higher segmentation performance was achieved using 2D and 3D U-Net. CoSinGAN can capture the conditional distribution of a single radiological image and synthesize high-resolution and diversified radiological images that closely fit the input conditions. The work's drawback is that the structural masks of the lungs and diseased regions must still be drawn by hand.

Deng et al. [21] developed lung infection segmentation network called “Inf-Net”. Infected region extraction usually faces problems such as infection extraction variation, low density contrast between the infected and normal region etc. Here, a parallel partial decoder generates a global map by aggregating high-level features. Explicit edge-attention and implicit reverse attention are used to model boundaries and improve representations. The development of a semi-supervised segmentation framework named "Semi Inf-Net" removed the limitations of CT images with segmentation annotations. For COVID-19 infection segmentation on CT images, a domain adaptation based self-correction model (DASC-Net) is proposed in [22], which consists of a novel attention and feature domain enhanced domain adaptation model (AFD-DA) to solve domain shifts and a self-correction learning process to refine segmentation results. An image-level activation feature extractor with a focus on lung anomalies and a multilevel discrimination module for hierarchical feature domain alignment are among the new features in AFD-DA. Even though this model outperformed "Semi Inf-Net", it faces limitation that, they presumptively annotated all of the source data samples. However, the number of well-annotated data samples was restricted, and DA approaches' performance can suffer significantly when there are fewer labeled examples.

### Severity prediction approaches

Majority of works focused on COVID-19 classification from normal CT images only. But once identified with the disease it is equally importance to get the severity level prediction. Mahdavi et al. [23] utilized patients’ clinical, laboratory, and demographic features at time of hospital admission to predict mortality prognosis, as these data can reduce the rate of mortality by prioritizing appropriate treatments. They implemented three ML models, using an SVM framework with three groups of input data. The first group of input data included demographic and clinical features; the laboratory features were in the second set, and the third set comprised a combination of both inputs. The criteria used for severity classifications were saturation of peripheral oxygen (${SPO}_{2}$) and respiratory rate (RR). ${SPO}_{2}$ of less than 90 and an RR greater than or equal to 30 were categorized as severe cases. Moreover, the authors stated that non-invasive (clinical and demographic) features are able to give a better prediction of mortality even when there are fewer of them.

In [24], they collected data from 641 patients and developed a model that calculates risk-score to predict intensive care unit (ICU) admissions and mortality rates. The authors also identified the key clinical features to be considered for ICU admission and mortality prediction. A reduced lymphocyte count was amongst the top predictors of ICU admission, as was history of smoking. The authors also validated the developed risk-score model with different internal datasets. Su et al. [25] used another dataset of 93 mild and 32 severe cases of COVID-19 to develop progression to severe symptoms prediction model. The model achieved 94.1% sensitivity and 90.2% specificity, and was under the ROC curve (AUC) of 94.4%. Although the authors found that 17 features could be used to distinguish between mild and severe cases, they identified that only four such features were independent and plays key role in severity prediction that includes, C-reactive protein test (CRP), RR, comorbidities and lactate dehydrogenase (LDH). In [26], we observed that CT scores were manually calculated by evaluating the lobar involvement in chest CT, incorporating different clinical and laboratory features. However, these works employed clinical measures to obtain the risk score which required human intervention. After reviewing these works, we decided to develop an automatic severity prediction model along with COVID-19 diagnosis.

CT images chosen over X-Rays to develop the framework after reviewing the works, since CT image contains majority of the COVID-19 infection findings clearly even from the primary stages. Many of the developed models faced generalization issues due to the dataset limitations. Hence, preferred largest publicly available dataset of COVID-19 CT images which was collected from different cohorts so that the model could be incorporated into real-time CAD applications. But the conventional segmentation approaches will not work due to the diversity in database. Hence, we opted semantic segmentation using DL. Majority of the reviewed works used either DL features or scale space variant features in their model development. Hence, we decided to develop our model using features that are local and scale, space, distortion, noise invariant to make use of COVID-19-related findings from each CT image, irrespective of the diversity in database. Since direct feature engineering involved in classical ML, these algorithms are quite easy to interpret and understand. Hence, we finalized with classical ML approaches for classification. To fill the research gap in COVID-19 diagnosis application, it was necessary to get an infection segmentation model along with an automatic disease severity prediction. To get a precise infection segmentation model even under real-time, DL semantic segmentation concept implemented. In total, the framework will give a complete automatic COVID-19 real-time CAD model along with infection extraction, severity score prediction and disease stage identification.

## Methods

This section contains information on all the materials we used, description of processes as well as the methodologies used to create the COVID-19 classification and infection segmentation architecture.

### Description of materials

#### CT-scan database

In this research, we used datasets from the China National Centre for Bioinformation [27], which provides a large CT image dataset. In this dataset, COVID-19 is referred as novel coronavirus pneumonia (NCP). The images in this collection were compiled from the China Consortium of Chest CT Image Investigation cohorts (CC-CCII). They also provided the metadata which includes patient ID, scan ID, liver function, lung function, age, sex, critical illness and time of progression. CT images and metadata mentioned, were acquired at the time of their hospital admission. Across the entire dataset, CT images vary in size from 256 × 256 till 2592 × 2592 and are in “jpg” and “png” formats. In addition to the complete set of CT images from different categories, they also provided information regarding 55,692 CT images with lesions belonging to both NCP and common pneumonia (CP) in one of the csv files named “lesions_slices.csv”. Moreover, it provides a dataset of 750 CT images obtained from 150 patients with manual pixel annotations by radiologists, provided by another study [28] which used the same dataset. In the pixel-labelled images, the pixels are annotated as zero for background, one for lung field, two for GGOs, and three for consolidation (CL). The complete details of the abovementioned dataset are mentioned in Table 1.

**Table 1:** CT image details of complete database for two classes

Category	NCP	Normal
Total number of patients	929	818
Total number of scans	1544	1069
Total number of CT slices	156,071	92,853
Number of CT slices with lesions	21,872	NA*
Data size	17.4 GB	9.1 GB

NA*: not available

#### SIFT, SURF, and ORB techniques

Both global and local features exist. Global features describe images as a whole, including their contour, texture, HOG features, etc., whereas local features give information regarding each keypoint in the image, such as SIFT, SURF, ORB, etc. In today’s medical imaging applications, SIFT [29], SURF [30] and ORB [31] techniques are also widely used in the feature extraction stage. As they are scale space invariant and robust against distortions, noise, etc., any kind of deformation present in CT images will not affect the CAD system diagnosis if we train a system using them. In these techniques, keypoints were identified from the ROI, and descriptors of these key points were generated. The performance of SIFT is close to real-time performance. Four key stages of SIFT feature extraction method includes, scale-space extrema detection, keypoint localization, orientation assignment, and keypoint descriptor. Here, keypoint description is constructed by taking into account the 16 × 16 neighborhood surrounding the keypoint, which is partitioned into 16, 4 × 4 subblocks, each with an 8-bin orientation histogram. Hence, if N keypoints are detected from the input CT image lung region using SIFT, it provides a descriptor of size N × 128.

SURF is similar to SIFT but offers faster computation, making it suitable for real-time applications. The two main steps of SURF are feature extraction and feature description. The detector is based on the Hessian matrix, while descriptor describes the distribution of Haar-wavelet responses in the vicinity of the interest point. The feature description stage consists of two steps: first, fix a reproducible orientation based on information from a circular region surrounding the keypoint, and then construct a square region centered on the keypoint that is oriented along the previously determined orientation. Then, the region is divided into 4 × 4 subregions. A few simple features are computed for each sub-region at 5 × 5 regularly spaced sample points. A four-dimensional descriptor vector is provided for each sub-region that includes $\sum dx$,$\sum dy$, $\sum \left|dx\right|,$ and $\sum \left|dy\right|,$ where $dx$ and $dy$ are Haar wavelet response in the horizontal and vertical directions, respectively. Hence, if there are N keypoints, we obtain an N × 64 feature descriptor. ORB is created from the Fast [32] (Features from Accelerated and Segments Test) keypoint detector and the BRIEF (Binary robust independent elementary feature) [33] descriptor. Keypoint "p" is found by comparing the brightness of that pixel to the 16 pixels surrounding it in a tiny circle. It is chosen as a keypoint if it is darker or brighter than p by more than eight pixels. ORB adds orientation assignment, such as left or right facing, using a multiscale image pyramid based on how intensity levels fluctuate around a keypoint as identified by an intensity centroid. BRIEF takes the keypoints and converts them into binary feature vectors (binary feature descriptors).

#### DeepLabv3+ architecture

Here, two DL models were developed for two-class and three-class segmentation. During the initial development stages of the CAD system, a two-class segmentation network using DeepLabv3+ was used for the segmentation of lungs from the CT image because in the later stages of ML, features were extracted from the lung regions only. Once the patient CT scan image is diagnosed with COVID-19 in our CAD system, the corresponding CT image is passed into the three-class segmentation DL model. The model was developed using DeepLabv3+, with its weights initialized using pre-trained networks. In three-class segmentation, the three classes indicate background, lung field, and infected regions (GGO, CL, etc.).

Google developed DeepLabv3+ by introducing a simple but effective encoder-decoder structure which is capable of performing much better semantic segmentation, especially along the object boundaries. This feature has led to its widespread use in medical image segmentation tasks using DL. The developers combined significant features from atrous spatial pyramid pooling (ASPP) and encoder-decoder structure. ASPP always promotes the encoding of multilevel contextual information, whereas an encoder-decoder structure captures the sharp object boundaries. The encoder section consists of atrous separable convolution, combination of atrous depthwise and pointwise convolution. This feature helps to reduce computational complexity by maintaining the same performance or even achieving a better performance. When atrous convolution is applied over an input feature map of “i” with convolutional filter “w” at atrous rate “r”, the output feature map “o” at each location “x” is given by Eq. 1.

1 $$o\left[ x \right] = \mathop \sum \limits_{k} i \left[ { x + r \cdot k } \right] w\left[ k \right]$$

**Fig. 2:**
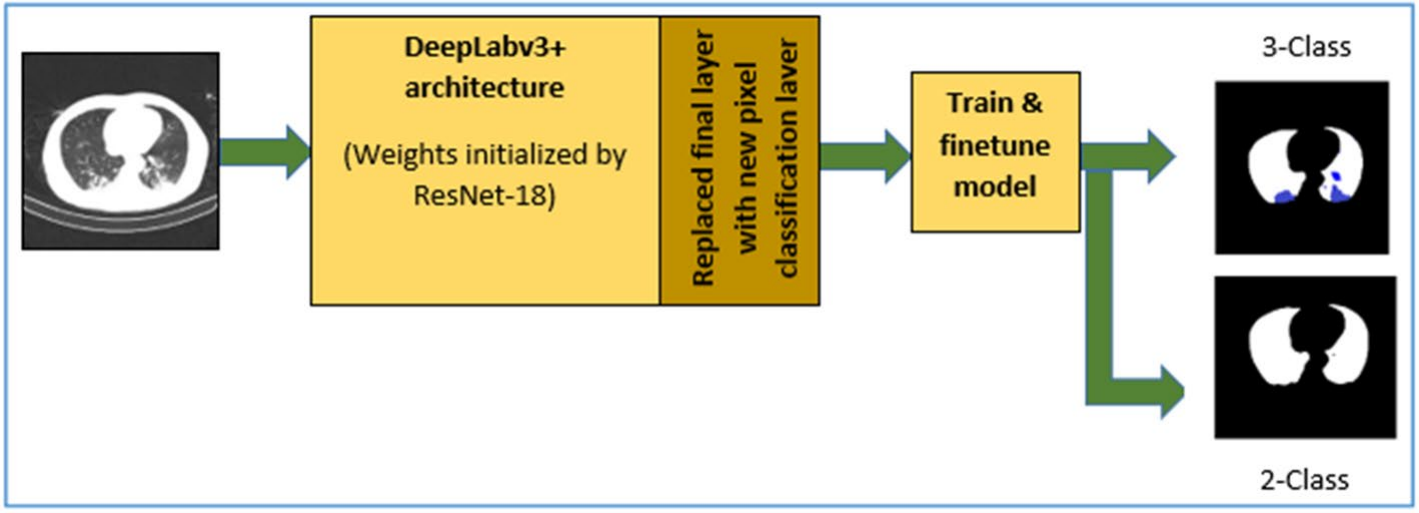
Semantic segmentation using DeepLabv3+

**Fig. 3:**
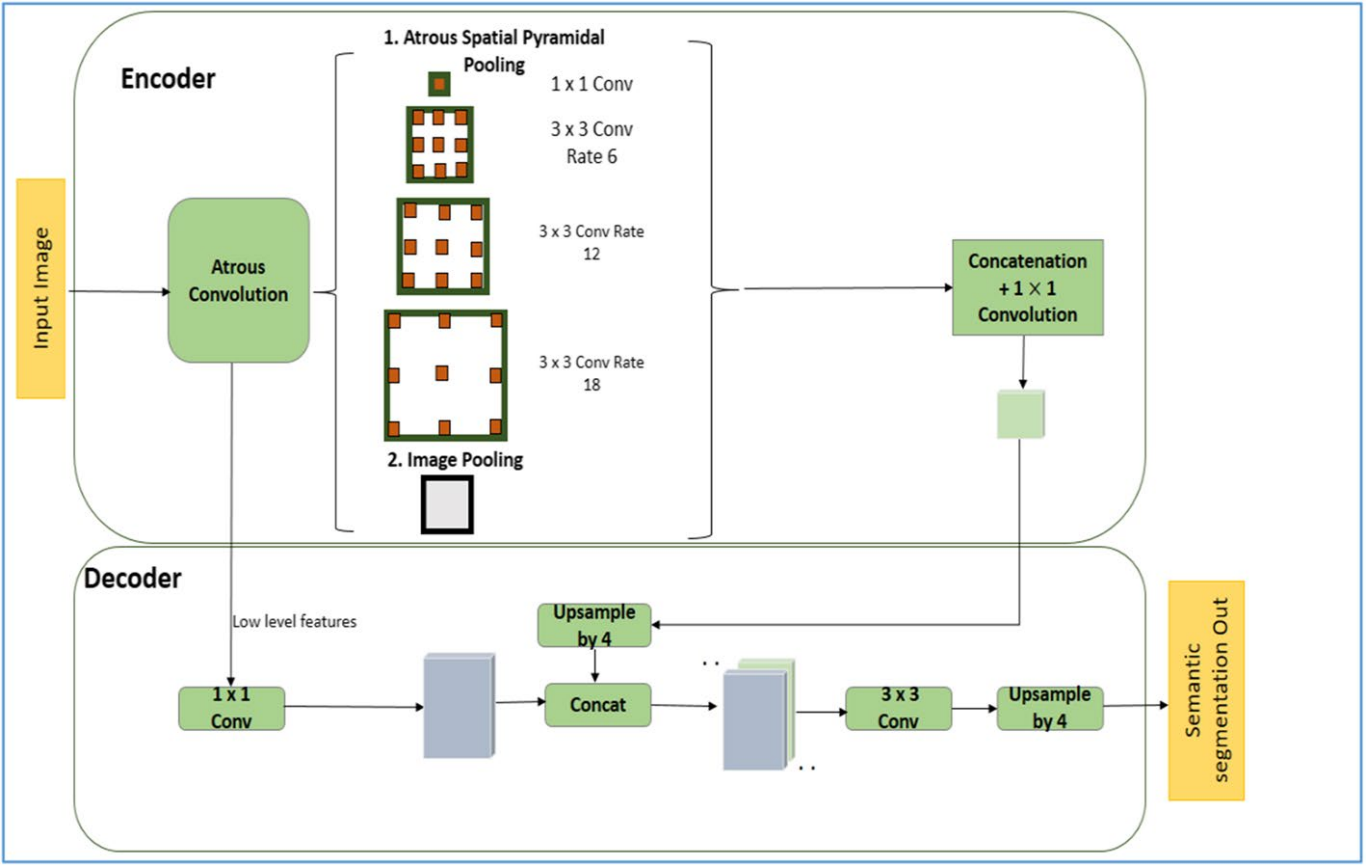
DeepLabv3+ architecture

To perform semantic segmentation, an output stride of 16 was chosen. By varying atrous convolution rates, the filter fields of view can be varied, and rich contextual information can be achieved. In the current study, the different atrous rates involved were 1, 6, 12, and 18. The decoder module is responsible for the detailed object boundary recovery. Bilinear upsampling of encoder features was performed by factor of four, and low-level features were concatenated. Then, 3 × 3 convolutions were applied followed by another simple bilinear upsampling by a factor of four. Low-level features typically have a greater number of channels. Hence, before concatenation a 1 × 1 convolution was applied to them for the number of channels reduction. Since DeepLabv3+ applies atrous separable convolution to encoder and decoder module, the network performs faster and more efficiently in segmentation tasks than other deep neural networks. The architecture of DeepLabv3+ deep neural network used for automated lung segmentation is shown in Figs. 2 and 3.

#### ML classification

In order to obtain a finetuned model, we adopted hyperparameter tuning by grid search (GS) and cross-validation concepts. A GS is used to get model optimal hyperparameters by an exhaustive search through a manually selected subset of the hyperparameter space that results in the most accurate predictions. For each classifier, we set a specific range of values for hyperparameters and finetuned by GS method. Using K-fold cross-validation, the dataset is divided into k number of subsets (folds) then trained all the subsets apart from one (k − 1), which was used to test the model. This same process was repeated k times, with each iteration reserving a different subset for testing. In this way, the model obtained the correct patterns from the data. Here performed five-fold cross-validation by shuffling the data each time.

#### Infection region segmentation

COVID-19-infected regions were segmented through DL semantic segmentation using CT images and their corresponding ground truths, and infected regions were labelled. To obtain the best infection segmentation model, the network was finetuned by varying maximum number of epochs, minimum batch size, optimizer, etc. DL semantic segmentation model can be evaluated using measures like pixel accuracy, IoU, BF score etc. Pixel accuracy is the proportion of correctly identified pixels to the entire number of pixels independent of class (Eq. 2). IoU is the ratio of the number of correctly classified pixels over the number of ground truth pixels and the predicted pixels in that class (Eq. 3) and BF score indicates how closely each class's predicted boundary aligns with its actual boundary (Eq. 4).

2 $$Pixel{ }Accuracy = { }\frac{TP + TN}{{TP + TN + FP + FN}}$$

3 $$Score = { }\frac{{2 \times precision{ } \times recall}}{{precision + { }recall}}$$

4 $$IoU{ }score = { }\frac{TP}{{{\text{TP }} + {\text{ FP }} + {\text{ FN}}}}$$

where TP—True Positive, TN—True Negative, FP—False Positive, and FN—False Negative.

### Design and setting of the study

**Fig. 4:**
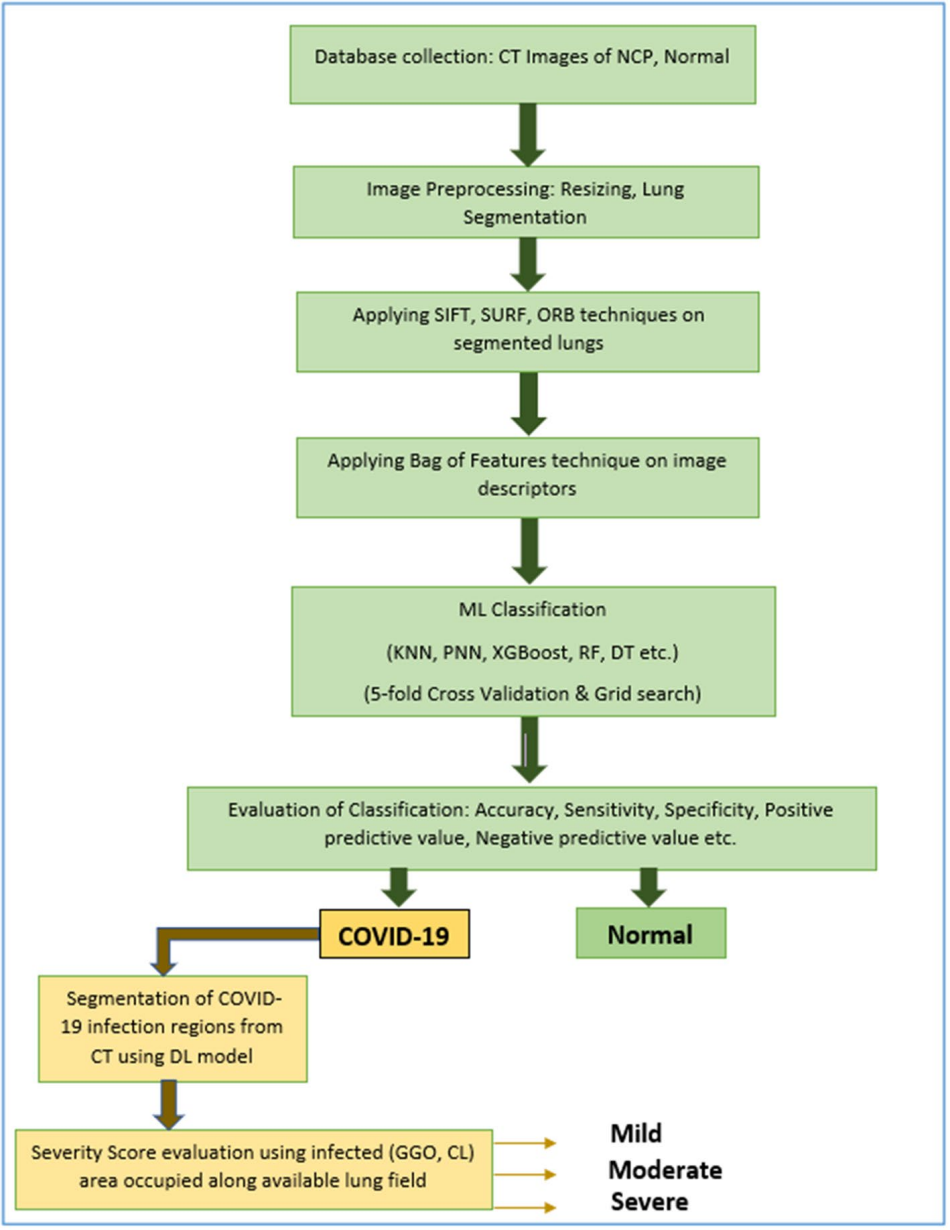
Proposed methodology

The proposed method used for the COVID-19 classification and infected region segmentation model is illustrated in Fig. 4. The system was built by extending the classification model with a semantic segmentation framework, using DL for COVID-19-infected region extraction along with severity calculation and disease stage prediction.

The low resolution offered by X-rays causes difficulties in identifying GGOs, crazy-paving patterns, and other COVID-19-pneumonia-specific indications that help to differentiate the condition from viral pneumonia. CT scan is more accurate for a COVID-19 diagnosis and it can identify the exact problem location more precisely. Hence, chose CT scan dataset for the proposed model development. To achieve generalization capability and to implement in high performance real-time CAD systems, selected the largest publicly available database that comes from different hospitals that are performed under different conditions and using different CT machines, which will have improved its diversity. Among the complete set of CT images mentioned in Table 1, finalized 6,000 CT images of chest CT scans, of which 3,000 belonged to COVID-19-affected patients and 3,000 belonged to normal people. The selection was based on the metadata information provided along with the database. The database chosen for experimentation is not biased, because it is constructed purely based on the ground truth, i.e., COVID-19 affected region containing CT slice information is already available in the ground truth file. And hence only those slices are selected without any repetition. Also, some patients had more than one scans under certain time gap. During this time, the patient chest CT scan may show huge variations with new COVID-19 infection patterns which is necessary for training the model. CT scan of each patient was available as CT slices within the scan folder which was already in 2D format. After analysing the slices, selected the slices which are effective to develop the model framework. For example, the initial, final CT slices will not include much information regarding the lung cross sections. Also, in between certain slices will be sharing the same information.

While validating the finalized set of CT images, noted that they varied in size from 256 × 256 to 2592 × 2592. Hence, the complete finalized CT images were resized to 256 × 256 with bilinear interpolation. As mentioned earlier, the diversity in databases can be a challenge in the lung segmentation stage. Conventional segmentation algorithms face limitations such as difficulty in selecting the optimal threshold/hyperparameters; assumptions and approximation in selecting the parameters; lack of intelligence in understanding the image descriptions; and the fact that segmentation is performed with selected number features. Hence, it is better to perform segmentation using CNNs as semantic or instance segmentation. However, we preferred semantic segmentation because multiple objects of the same class are treated as a single entity rather than considering them as distinct individual instances, as is the case in instance segmentation. 2-class segmentation was used to segment the lung region from its background.

Among different semantic segmentation networks such as, SegNet, U-Net, DeepLabv3+ and FCN, DeepLabv3+ is one of the most recent fast and computationally efficient semantic segmentation network. It is capable of performing much better semantic segmentation, especially along the object boundaries due to its effective encoder-decoder structure. To use DeepLabV3+ network in a lung segmentation task, we tested it with five different pre-trained networks, namely ResNet with 18 and 50 layers, Inception-ResNet-v2with 164 layers, MobileNet-V2 with 53 layers, and Xception with 71 layers. We finally chose ResNet-18 for two-class segmentation, based on the experimentation explained in section five. Using the GS method, the optimal hyperparameters were selected based on the best performance for lung segmentation.

The segmented lung images from the DL model were taken, and SIFT, SURF, and ORB methods were preferred in the feature extraction stage to calculate the local features from the lung region. It gives information regarding each keypoint in the CT image unlike the global features that describe images as a whole, including their contour, texture, HOG features, etc. Also, the other handcrafted features are not invariant with scale, space, distortions, artifacts etc. which makes the developed system unstable with real-time CAD applications. First, the keypoints were located from the segmented lung image, after which descriptors were created. Each keypoints from these methods are valuable to obtain the critical information regarding the CT image in taking COVID-19 diagnosis decisions. If there were “K” keypoints in one such image, SIFT provided a descriptor array of size K × 128, K × 64 from SURF and K × 32 from ORB. However, the number of keypoints in each image varied, based on the image details available in them. Each image was considered sequentially, and a new descriptor was appended to the descriptor of the previous image.

The appended descriptor array could not be fed directly into the classifier due to its high dimensionality. We needed to arrange the features and transform them into an appropriate format so as to reduce the dimensionality and make it computationally efficient. This was done by using the bag of features (BOF) technique [34]. After extracting the descriptors using the SIFT, SURF, and ORB methods, we used clustering algorithms to cluster these feature vectors. The most commonly used clustering algorithm is k-means, which tries to cluster into k clusters, whereby points are part of the cluster corresponding to the closest cluster center. The clustering criteria consists of minimizing the sum of square distances between the cluster center and the points that belong to it. After the clustering was completed, we obtained a dictionary composed of k vectors, called visual words. We were able to find visual words from the dictionary that depicted each SIFT feature of the image. The result was the creation of a k-dimensional histogram, which represented the SIFT feature of the image. The discrimination between COVID-19 and normal groups of CT features was evaluated with an analysis of variance (ANOVA), and a box plot analysis was used to analyze the characteristics of features used in ML classification. Box plots include the basic statistical features like minimum, maximum, standard deviation, 25% quartile, 50% quartile, and 75% quartile. Using the box plot, we can identify features that help distinguish diagnoses. Feature reduction methods were not implemented here since each keypoints are valuable in giving the CT image information. Feature reductions may affect these keypoints and sometimes we may miss the critical information in taking COVID-19 diagnosis decisions. But the above-mentioned methods were easy to implement and faster.

The classification phase was concerned with classifying the features obtained from the previous stages and detecting whether the segmented lung region was normal or abnormal. To use in real-time CAD applications, the framework should be faster. Classical approaches aren’t so computationally expensive, one can also iterate faster and try out many different techniques in a shorter period of time. Due to the direct feature engineering involved in classical ML, these algorithms are quite easy to interpret and understand. In addition, tuning hyper-parameters and altering the model designs is more straightforward since we have a more thorough understanding of the data and underlying algorithms. On the other hand, deep networks are very “black box” in that even now researchers do not fully understand the “inside” of deep networks. The classifiers applied in the present work are RF, K-nearest neighbors (KNN), eXtreme Gradient Boosting (XGBoost), decision tree (DT), and probabilistic neural network (PNN) etc. We preferred GS technique to fine tune the model since it is easy to implement and understand and also to robust the prediction power. In this, we simply build a model for every combination of various hyperparameters and evaluate each model. For each parameter, possible set of values was available for all the classification algorithms we implemented. Here, ‘GridSearchCV’ from sklearn is employed to use the GS algorithm to detect the optimal hyperparameters. The MATLAB software (version R2021a, MathWorks Inc) and Python 3.7 with Pycharm IDE were used to implement the ML process.

### Deep learning model for infection region extraction

**Fig. 5:**
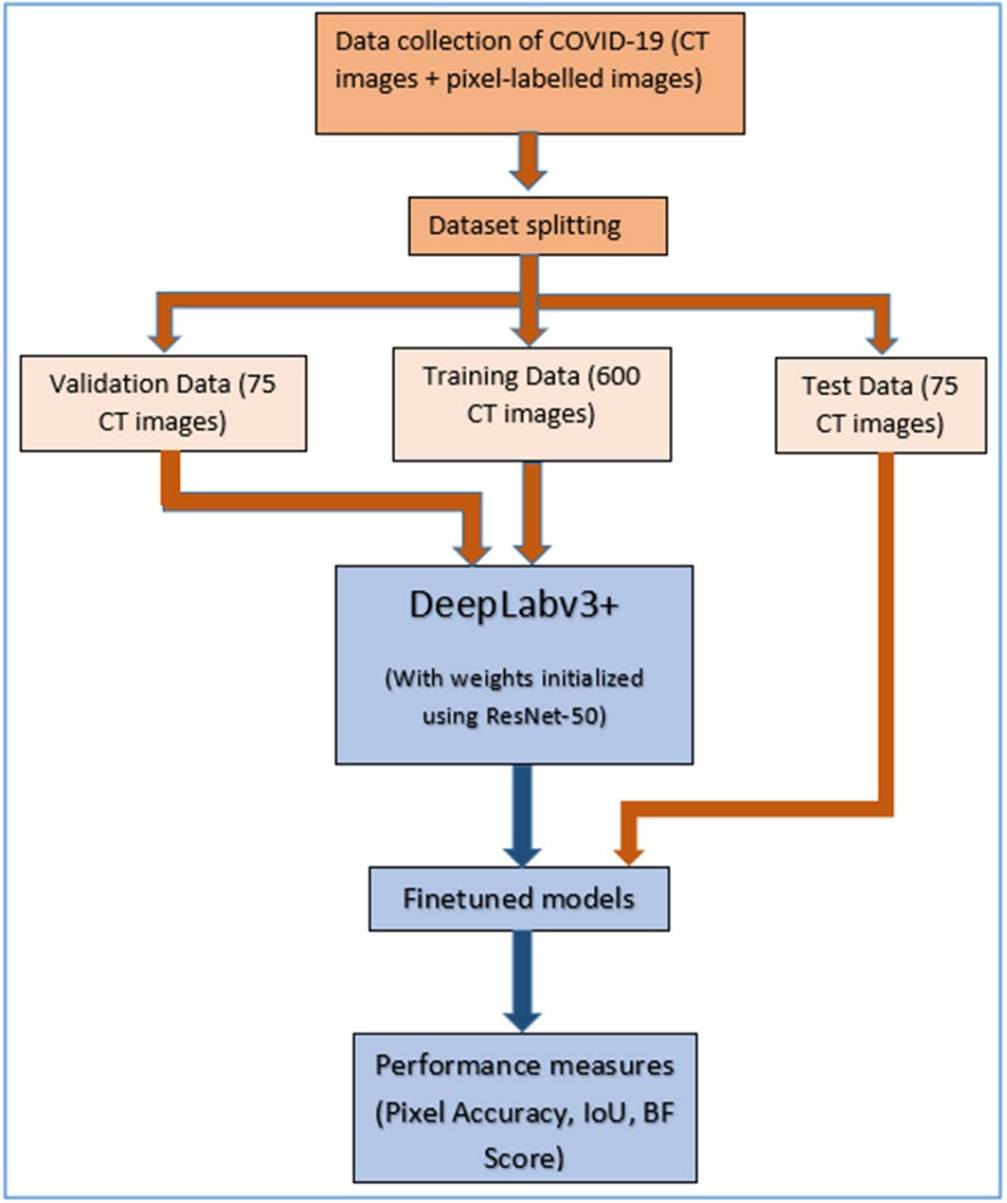
Workflow of infected region extraction using deep learning

The workflow proposed in the development of the semantic segmentation network for infected region extraction using DL is shown in Fig. 5. As mentioned before, due to the diversity of database, we preferred semantic segmentation model using DL to get the infection region extraction model. Also, COVID-19 infected regions will be identified with the CT findings such as GGO, consolidations etc. which could be precisely extracted with accurate boundaries only by using DL semantic segmentation networks. In addition, we had 750 CT images of COVID-19 patients showing diverse infection patterns along with pixel labelled ground truths of infected regions which was sufficient to train the model. Among various networks we selected DeepLabv3+ due to its computational efficiency and speed. Also, it is capable of performing much better semantic segmentation, especially along the object boundaries due to its effective encoder-decoder structure. The three-class segmentation was used for infected region extraction from the CT images of COVID-19-diagnosed patients.

Infection region extraction helps to identify the severity of disease. The 750 CT images and their corresponding 750 pixel-labelled images annotated by radiologists used in the DL semantic segmentation had size of 512 × 512. All CT images were resized to 256 × 256 to enable a better performance with less training time. We divided the dataset and its corresponding pixel-labelled images in the ratio of 80% for training and 10% each for validation and testing. The model takes more computational power and time to run while using multi-fold cross-validation during segmentation model training task. Moreover, the training data covers most of the mild, moderate and severe infections possibilities over the lungs from various patients and hence it could learn well from these training samples. Hence here we preferred data splitting instead of multi-fold cross-validation. However, since we needed to focus on infected region segmentation, the pixel-labelled images were annotated as two for COVID-19-infected regions (GGO, CL, etc.) in addition to background and lung fields.

We used the concept of transfer learning since it reduces training time drastically and also gives better models, even when small training sets are used. We experimented DeepLabv3+ architecture, using different pre-trained networks, ResNet-18, ResNet-50, and MobileNet-V2 by shuffling the dataset each time. We also performed hyperparameter tuning over the network during the model training for the parameters, ‘max epochs’, ‘initial learning rate’, ‘optimizers’, ‘min. batch size’ etc. to improve the model performance. The maximum number of epoch values changed between 20, 50, and 100, the minimum batch size between 8, 16, and 32, and the initial learning rate between 0.001 and 0.0001. We also changed optimizers, stochastic gradient descent with momentum (SGDM) and Adam. The network giving the best IoU values chosen as the best segmentation model for COVID-19 infection region extraction. The proposed framework was implemented on a desktop computer with Intel i7, an 11th generation processor with a speed of 2.50 GHz of eight cores with 16 GB DDR4 RAM and Windows operating system. The system was also equipped with single graphical processing units (GPUs) of the GTX 1660 SUPER 6 GB graphics card to accelerate the computation process.

## Results

Here we present the experimental results of various stages of our proposed model. Input CT images of COVID-19 and normal cases as well as various image pre-processing results are illustrated in Table 2.

**Table 2:**
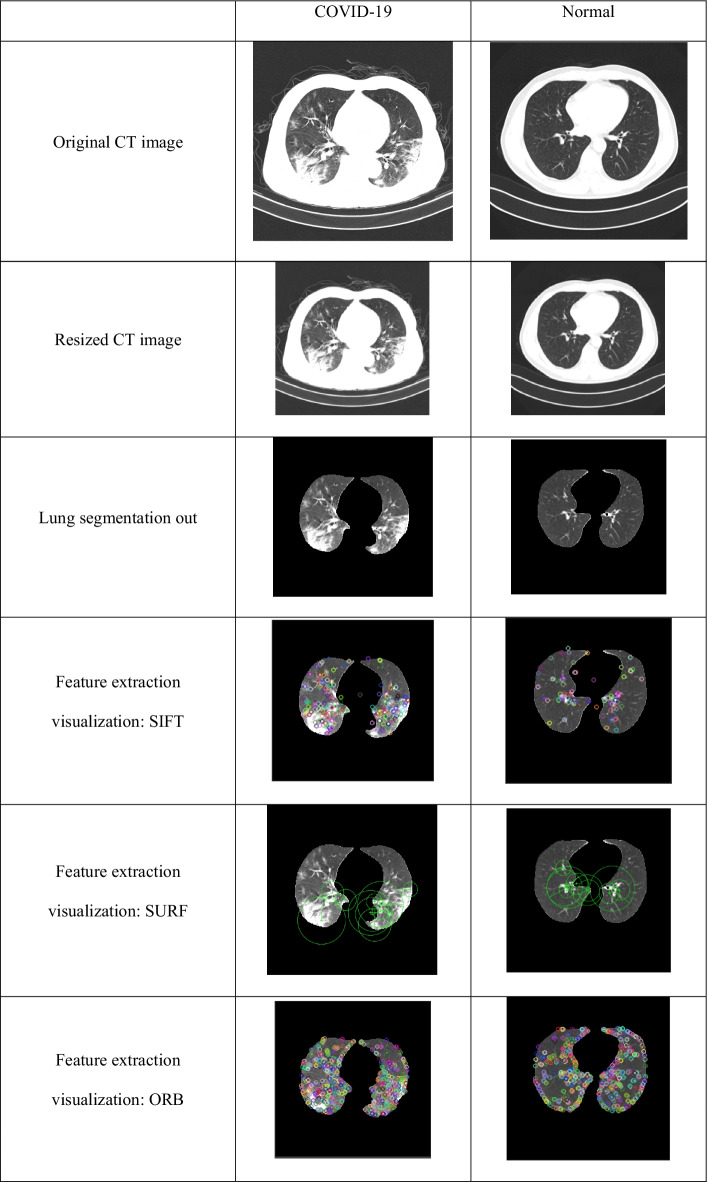
Pre-processing results of COVID-19 and normal CT images

Lung segmentation was carried out using DL semantic segmentation. A two-class segmentation model was developed for the segmentation of lungs from the background regions of CT images. ‘DeepLabv3+’ with weights initialized from different pre-trained networks were experimented. Table 3 shows class metrics of the experimentation tasks. After experimentation, DeepLabv3+ with ResNet-18 as a pre-trained network with maximum epochs of 100, minimum batch size 16, initial learning rate 0.001 and ‘adam’ optimizer was chosen as the best two-class semantic segmentation model since it provides the highest segmentation performance measures for the lung field class.

**Table 3:** Class metrics of two-class semantic segmentation using DeepLabv3+ with weights initialized by different pre-trained networks

Pre-trained network	Mini batch size	Accuracy	IoU	Mean BF score	Accuracy	IoU	Mean BF score
		(Background)			(Lung field)		
ResNet-18	8	0.9977	0.9966	0.9939	0.9921	0.9771	0.9828
**ResNet-18**	**16**	**0.9977**	**0.9968**	**0.9947**	**0.9939**	**0.9784**	**0.9852**
ResNet-18	32	0.9981	0.9952	0.9908	0.9804	0.9679	0.9738
ResNet-50	8	0.9978	0.9962	0.9924	0.9885	0.9722	0.9789
ResNet-50	16	0.9971	0.9946	0.9885	0.9818	0.9607	0.9656
MobileNet-v2	8	0.9973	0.9964	0.9949	0.9937	0.9744	0.9849
MobileNet-v2	16	0.9985	0.9960	0.9900	0.9818	0.9711	0.9712
MobileNet-v2	32	0.9983	0.9966	0.9925	0.9878	0.9757	0.9775

Bold usage is preferred to enhance the experimental result which provides the high performance

**Fig. 6:**
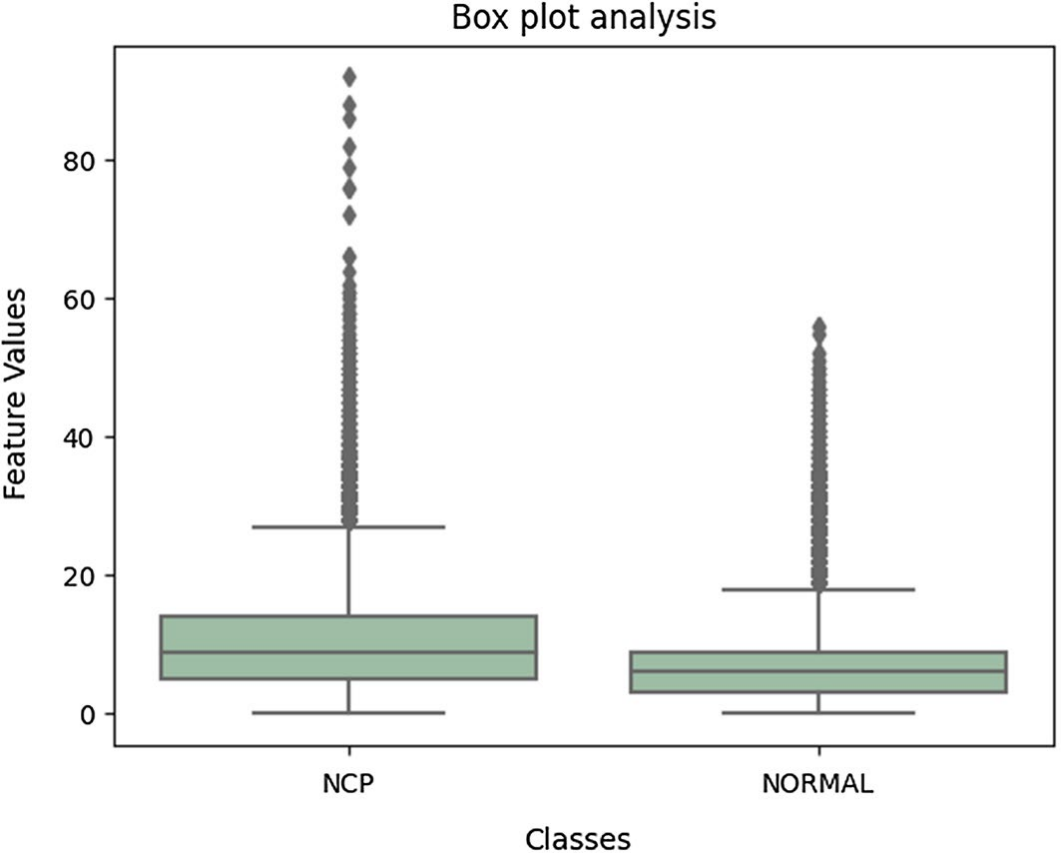
Boxplot for ORB features from 6000 CT images

In the feature extraction stage, 6,000 segmented lung regions of both COVID-19 and normal CT images were subjected to SIFT, SURF, and ORB methods, and the results were exported to Excel files. The feature sizes are shown in Table 4. The parameters used in SIFT, SURF, and ORB are described in Table 5. An ANOVA and a boxplot were used as part of the statistical analysis of features. Table 6 shows the results of the ANOVA and the boxplot obtained is visualized in Fig. 6 for ORB features.

**Table 4:** SIFT, SURF, and ORB feature sizes for COVID-19 and normal CT images

Feature extraction technique	COVID-19 feature size (3000 lung segmentation out)	Normal feature size (3000 lung segmentation out)
SIFT	448,368 × 128	205,670 × 128
SURF	266,775 × 64	137,820 × 64
ORB	1,048,576 × 32	1,048,576 × 32

**Table 5:** SIFT, SURF, and ORB parameters

SIFT		SURF		ORB	
Parameter	Value	Parameter	Value	Parameter	Value
nOctaveLayers	3	MetricThreshold	1000	scaleFactor	1.2f
contrastThreshold	0.04/0.03	NumOctaves	3	nlevels	8
edgeThreshold	10	NumScaleLevels	4	edgeThreshold	31
sigma	1.6	ROI	[1 1 size(I,2) size(I,1)]	firstLevel	0
				WTA_K	2
				scoreType	HARRIS_SCORE
				patchSize	31
				fastThreshold	20

**Table 6:** ANOVA results for SIFT, SURF, and ORB features

Method	Class name	Minimum	Mean	Quartile-1	Median	Quartile-3	Maximum	IQR
ORB	COVID-19	0	10.24	5	9	14	92	9
	Normal	0	7.11	3	6	9	56	6
SIFT	COVID-19	0	1.49	0	1	2	21	2
	Normal	0	0.69	0	0	1	26	1
SURF	COVID-19	0	0.89	0	0	1	29	1
	Normal	0	0.46	0	0	1	23	1

**Fig. 7:**
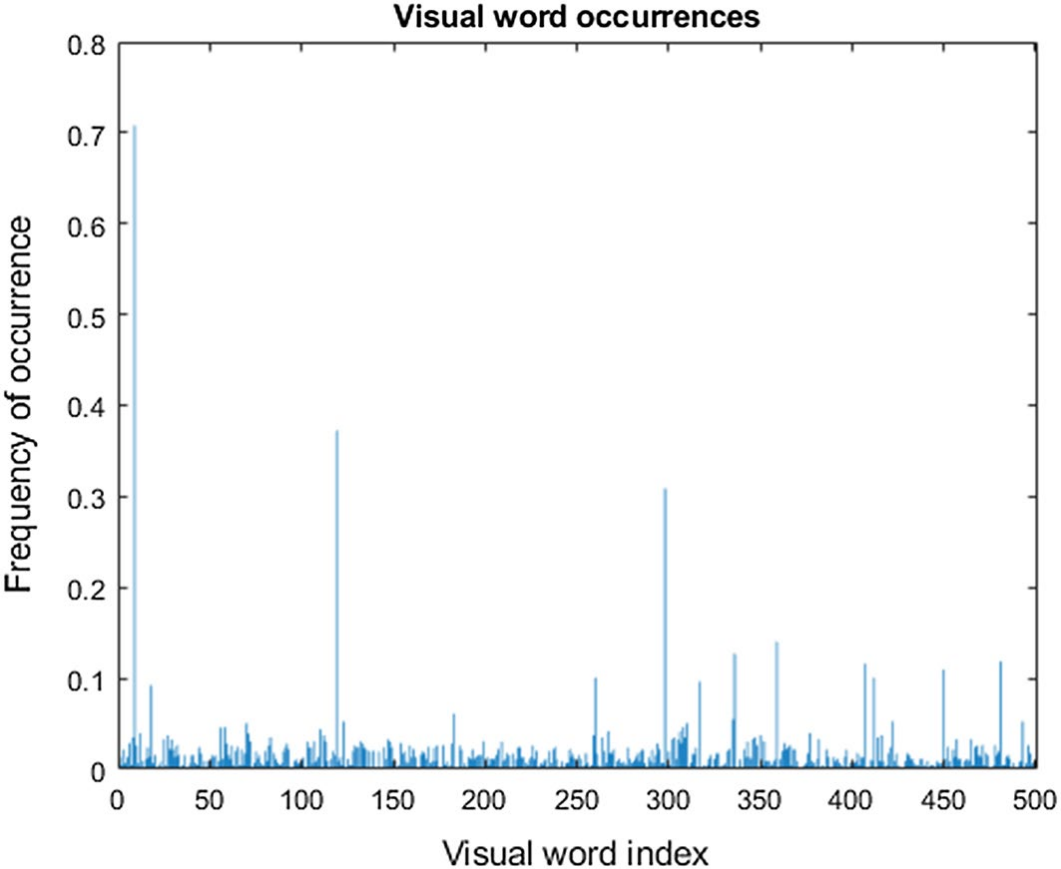
Histogram of visual word occurrences

Since the feature sizes varies, it cannot be directly fed into ML classifier for training the model. Hence bag of features technique is applied to cluster these features. This helps to have same feature size from each category and also dimensionality reduction could be achieved which will reduce the processing time. Using BoF, each image is represented by a feature vector of size "k", so each image is represented by a fixed-size vector. Since each image had a different number of keypoints, after descriptors were obtained from each image, they were concatenated vertically. Thereafter, we clustered all descriptors into k clusters using k-means. Each k cluster was associated with a centroid. These “k” centroids represented the main features of the whole training images and were called code words (visual words) which, together, made up the code book (visual vocabulary). In the next stage, descriptors were extracted using SIFT, SURF, or ORB methods from the test image and then converted into a feature vector of size “k”. First, a vector of “k” filled with zeros was created, where the *i*th element corresponds to the *i*th codeword (cluster). Similarly, for each descriptor the closest cluster was found. Finally, we created a vector that represented the frequency of codewords in the test image. This vector is known as the feature vector, and it can also be viewed as a histogram of features of the test image. A histogram of visual word occurrences for a sample set of 1500 images is illustrated in Fig. 7.

In the conventional ML classification stage, we performed five-fold cross-validation and hyperparameter tuning using the GS method. The hyperparameters used for each ML classifier are listed in Table 7.

**Table 7:** Classifiers and hyperparameters with value ranges

Classifier	Hyperparameter	Value range
Random Forest Classifier	Number of trees (n_estimators)	[1, 10, 50, 100]
	criterion	[‘gini’,’entropy’]
	max_features	[‘sqrt’,’log2’]
XGBoost	n_estimators	[100, 200, 300, 400, 500]
	learning_rate	[0.0001, 0.001, 0.01, 0.1]
KNN	n_neighbors	[3, 5, 11, 19]
	weights	[‘uniform’, ‘distance’]
	metric	[‘euclidean’,’manhattan’]
Decision Tree	max_leaf_nodes	list(range(2, 100))
	min_samples_split	[2, 3, 4]
	max_depth	np.arange(3, 10)
	criterion	['gini', 'entropy']
PNN	std	(0, 10)

The same classification was repeated using BoF from the SIFT, SURF, and ORB methods. The K value of K-means clustering also varied, with values of 50, 100, 500, 1000, 1500, 2000, 2500, and 3000 found. First, the classification using ORB features experimented with all these K values; for those which gave the best results, the remaining classification was carried out using SIFT and SURF features. The best results of ML classifiers using ORB, SIFT, and SURF features are given in Tables 8, 9 and 10, respectively. We then chose the PNN classifier with ORB features as the best classification model from its highest classification accuracy and least misclassification rate.

**Table 8:** Best classification performance measures for ORB features

Classifier	K	Class	PPV	NPV	Sensitivity	Specificity	Accuracy	MR*
XGBoost	2000	COVID	0.9930	0.9940	0.9940	0.9930	0.9935	0.0065
		Normal	0.9940	0.9930	0.9930	0.9940	0.9935	0.0065
RF	1000	COVID	0.9883	0.9880	0.9880	0.9883	0.9882	0.0118
		Normal	0.9880	0.9883	0.9883	0.9880	0.9882	0.0118
DT	50	COVID	0.9699	0.9684	0.9683	0.9700	0.9692	0.0308
		Normal	0.9684	0.9699	0.9700	0.9683	0.9692	0.0308
KNN	100	COVID	0.9983	0.9953	0.9953	0.9983	0.9968	0.0032
		Normal	0.9953	0.9983	0.9983	0.9953	0.9968	0.0032
PNN	100	COVID	**0.9987**	**0.9960**	**0.9960**	**0.9987**	**0.9973**	**0.0027**
		Normal	**0.9960**	**0.9987**	**0.9987**	**0.9960**	**0.9973**	**0.0027**

Bold usage is preferred to enhance the experimental result which provides the high performance

**Table 9:** Best classifier results for SIFT features

Classifier	K	Class	PPV	NPV	Sensitivity	Specificity	Accuracy	MR*
XGBoost	2000	COVID	0.9946	0.9777	0.9773	0.9947	0.9860	0.0140
		Normal	0.9777	0.9946	0.9947	0.9773	0.9860	0.0140
RF	1000	COVID	**0.9939**	**0.9812**	**0.9810**	**0.9940**	**0.9875**	**0.0125**
		Normal	**0.9812**	**0.9939**	**0.9940**	**0.9810**	**0.9875**	**0.0125**
DT	50	COVID	0.9709	0.9570	0.9563	0.9713	0.9638	0.0362
		Normal	0.9570	0.9709	0.9713	0.9563	0.9638	0.0362
KNN	100	COVID	0.9996	0.8881	0.8740	0.9997	0.9368	0.0632
		Normal	0.8881	0.9996	0.9997	0.8740	0.9368	0.0632
PNN	100	COVID	0.9974	0.8958	0.8840	0.9977	0.9408	0.0592
		Normal	0.8958	0.9974	0.9977	0.8840	0.9408	0.0592

Bold usage is preferred to enhance the experimental result which provides the high performance

**Table 10:** Best classifier results for SURF features

Classifier	K	Class	PPV	NPV	Sensitivity	Specificity	Accuracy	MR*
XGBoost	2000	COVID	0.9856	0.9840	0.9840	0.9857	0.9848	0.0152
		Normal	0.9840	0.9856	0.9857	0.9840	0.9848	0.0152
RF	1000	COVID	**0.9889**	**0.9841**	**0.9840**	**0.9890**	**0.9865**	**0.0135**
		Normal	**0.9841**	**0.9889**	**0.9890**	**0.9840**	**0.9865**	**0.0135**
DT	50	COVID	0.9505	0.9535	0.9537	0.9503	0.9520	0.0480
		Normal	0.9535	0.9505	0.9503	0.9537	0.9520	0.0480
KNN	100	COVID	0.9932	0.9324	0.9280	0.9937	0.9608	0.0392
		Normal	0.9324	0.9932	0.9937	0.9280	0.9608	0.0392
PNN	100	COVID	0.9961	0.9341	0.9297	0.9963	0.9630	0.0370
		Normal	0.9341	0.9961	0.9963	0.9297	0.9630	0.0370

Bold usage is preferred to enhance the experimental result which provides the high performance

MR*: Miss classification Rate

**Fig. 8:**
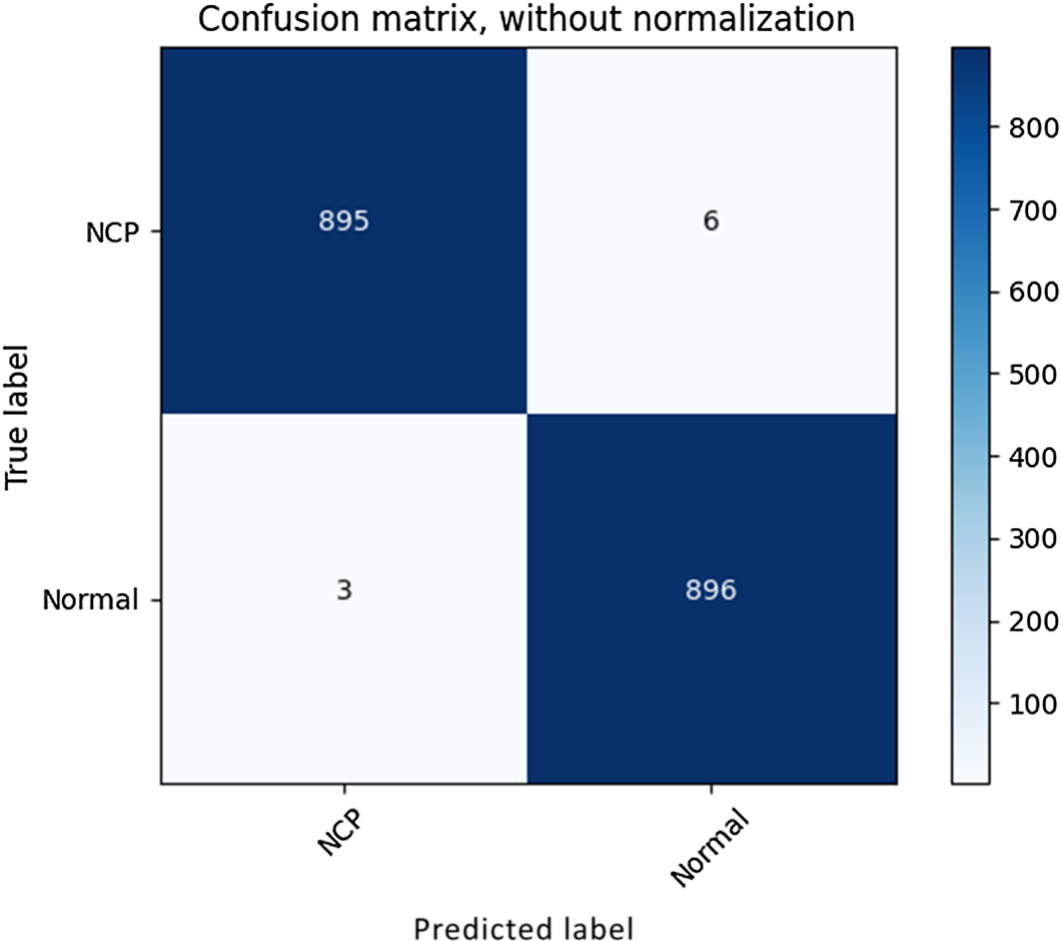
Confusion matrix of PNN classifier using ORB features

**Fig. 9:**
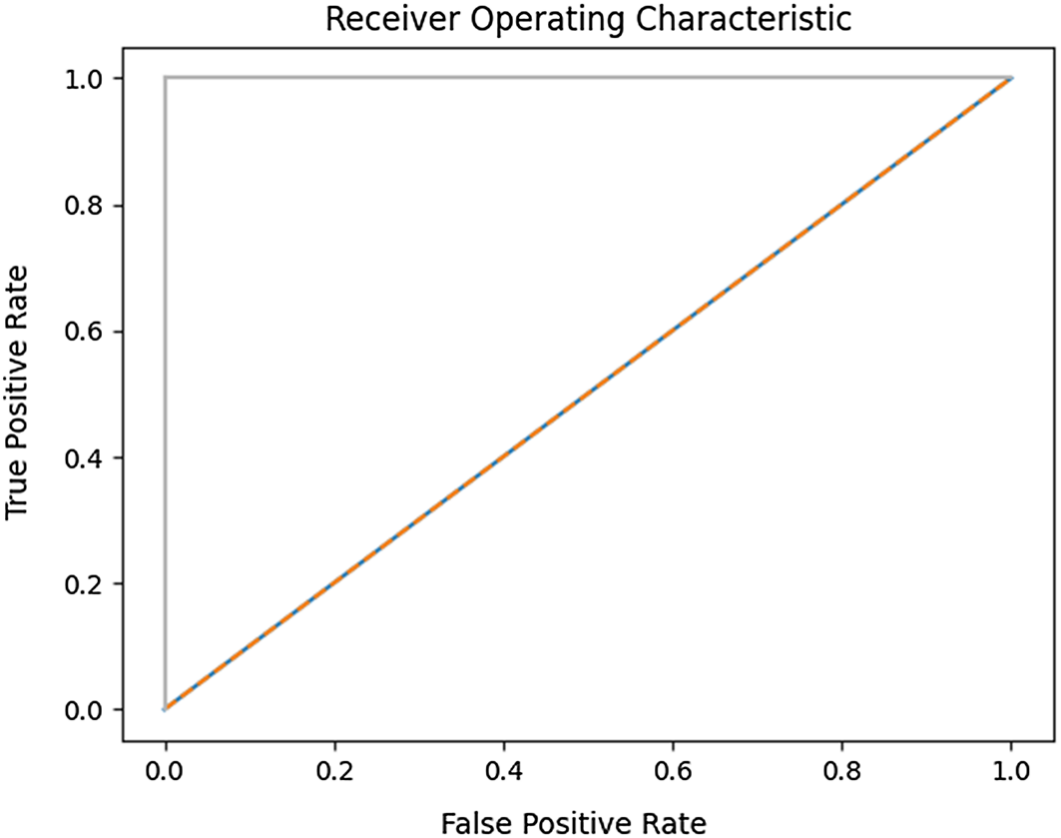
ROC curve of PNN classifier using ORB features

The confusion plot and ROC curve of the best classification model are shown in Figs. 8 and 9, respectively. We obtained an AUC value of 0.99888 from this.

If the input CT image was diagnosed with COVID-19, we then had to extract the infected region within the lungs. We employed semantic segmentation using DL to perform this task. The training was performed using 600 lung CT images and their corresponding pixel-labelled images with the help of DeepLabv3+ architecture. Similarly, 75 images were chosen for validation and the remaining 75 to test the trained model. Pixels were labelled as zero for background, one for lung field, and two for COVID-19-infected regions. Pixel distribution details for training, validating, and testing the dataset are presented in Table 11.

**Table 11:** Pixel distribution details of three classes

Class name	Training pixel counts	Validation pixel counts	Test pixel counts	Total number of pixels (%)
Background	1.3718e+08	1.7246e+07	1.713e+07	87.258
Lung field	1.8306e+07	2.1934e+06	2.2644e+06	11.578
Infected regions	1.79973e+06	221,827	266,203	1.163

**Fig. 10:**
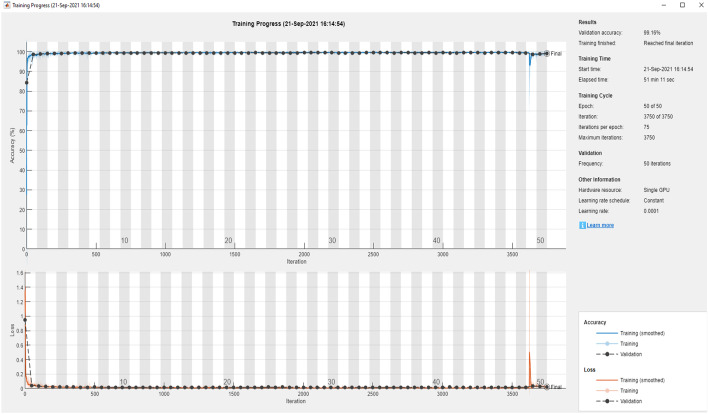
Accuracy and loss versus iteration plots of final DL semantic segmentation network

The semantic segmentation network weights were initialized using pre-trained networks such as ResNet-18 having 18 layers, ResNet-50 with 50 layers, and MobileNet-v2 with 53 layers. We used the concept of transfer learning to achieve better results with less training time. Using the GS method, the optimal hyperparameters were selected to obtain the model which performed best at infected region segmentation, as shown in Table 12. The best performance measures of each network model are specified in Tables 13 and 14. From these results, we selected DeepLabv3+ with its weights initialized using Resnet-50 since it provides the best segmentation measures for infection region class. Accuracy Vs iteration and loss Vs iteration visualizations for the fine-tuned, best CNN model are given in Fig. 10.

**Table 12:** Optimal hyperparameters used for infection segmentation

Parameter	Hyperparameters	Optimal value
Maximum epochs	20, 50, 100	50
Batch size	8,16,32	8
Momentum factor	0.9	0.9
Learning rate	0.001, 0.0001	0.0001
L2 regularization	0.0001	0.0001
Optimizers	‘sgdm’, ‘adam’	adam

**Table 13:** Best dataset metrics from each network after grid search

Network name	MBS*	ILR*	Global accuracy	Mean accuracy	Mean IoU	Weighted IoU	Mean BF score
ResNet-18	32	0.001	0.9936	0.9190	0.8798	0.9878	0.9210
**ResNet-50**	**8**	**0.0001**	**0.9947**	**0.9404**	**0.8968**	**0.9899**	**0.9453**
MobileNet-v2	8	0.001	0.9925	0.9307	0.8787	0.9859	0.9170

Bold usage is preferred to enhance the experimental result which provides the high performance

**Table 14:** Best class metrics from each network after grid search

Network name	MBS*	ILR*	Class name	Accuracy	IoU	Mean BF score
ResNet-18	32	0.001	Background	0.9992	0.9966	0.9949
			Lung field	0.9727	0.9522	0.9535
			Infection	0.7851	0.6905	0.7454
**ResNet-50**	**8**	**0.0001**	Background	**0.9989**	**0.9974**	**0.9951**
			Lung field	**0.9780**	**0.9594**	**0.9605**
			Infection	**0.8443**	**0.7338**	**0.7992**
MobileNet-v2	8	0.001	Background	0.9981	0.9965	0.9932
			Lung field	0.9699	0.9390	0.9455
			Infection	0.8240	0.7007	0.7636

Bold usage is preferred to enhance the experimental result which provides the high performance

ILR*: Initial Learning Rate, MBS*: Minimum Batch Size

From the finalized model, we achieved a mean IoU value of 0.8968 and a weighted IoU value of 0.9899. Using the trained model, we extracted the infected regions from each COVID-19 CT image. Based on the infection within the lungs, the disease stages were classified as mild, moderate, or severe, with the severity score evaluated using Eq. 5. The disease stage was classified as mild if the score was below 0.25, moderate if it was between 0.25 and 0.5, and severe if it varied between 0.5 and 1. Table 15 illustrates examples of COVID-19 CT images at different stages, visualization of the infected region extractions obtained by our model, and the corresponding severity scores.

5 $$Severity\,Score = { }\frac{Total\,area\,of \,infected \,regions}{{Area\, of\,lung\, field }}$$

**Table 15:**
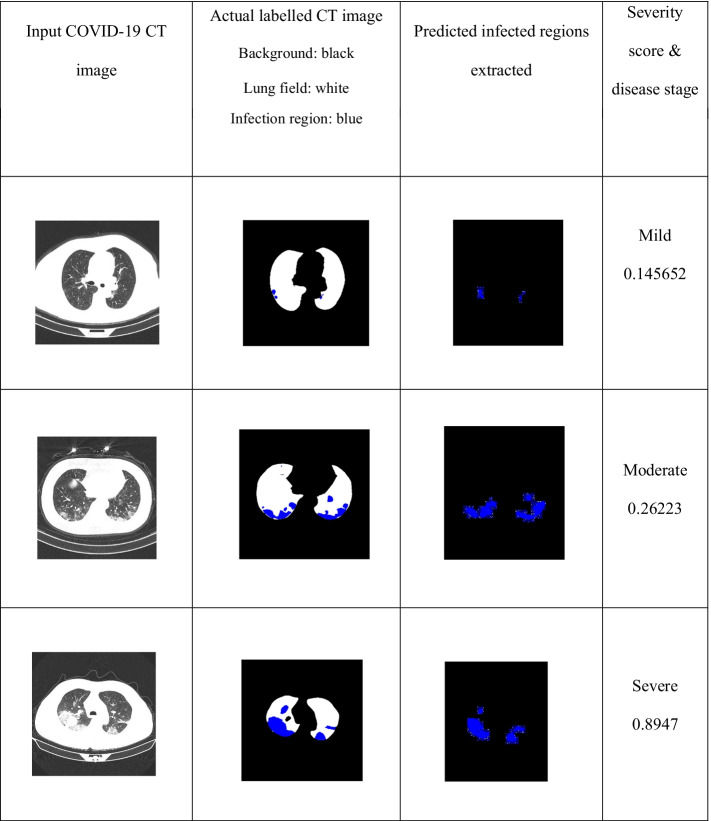
Infected region extractions; severity score of different COVID-19 CT images

## Discussion

Using the proposed framework, we were able to automatically detect whether the input chest CT scan image belonged to a COVID-19 patient or a normal case precisely. The implementation of SIFT, SURF, ORB image descriptors along with bag of features techniques helped to achieve the highest ML classification performances by its capability to differentiate chest CT regions with COVID-19 infections from normal CT accurately. If it was COVID-19, it could automatically extract the infected regions from the CT scan, calculate the severity score, and identify the disease stage as mild, moderate, or severe. Since all these tasks can be achieved by a computer, manual tasks can be eliminated and medical professionals can start treatment based on stage of disease without delay. Moreover, limited resources, such as the radiologists availability in some hospitals, can be effectively used. Table 16 shows a comparison of our proposed model for COVID-19 detection from chest CT images with some recent COVID-19 diagnosis works which used the same database. As can be observed, our model has the highest classification accuracy as well as infection region segmentation performance.

**Table 16:** Performance comparison table

Ref	Proposed method	Result	Limitation
[35]	Classification: COVID-CT-Mask-Net model, Segmentation: MaskR-CNN	Classification accuracy: 0.9166, sensitivity: 0.9080, specificity: 0.9210, F1-score: 0.9150	Poor model generalization capability
[36]	Classification using DL features from EfficientNet and clinical data	AUC = 0.8274	Not an automatic approach, collection of clinical data requires manual intervention
[37]	Classification: COVIDNet-CT, heterogeneous composition of conventional spatial, pointwise, depthwise convolution layers	Classification accuracy: 0.973, specificity: 0.999, PPV:0.99, NPV: 0.993	Architecture faces generalization issues, no infection extraction approach
[38]	Classification: VGG16 deep neural network + ensemble learning	Classification accuracy: 0.9357, specificity: 0.9393, sensitivity: 0.9421, precision: 0.894, and F1-score: 0.9174	Only experimented with VGG model, no infected lesion segmentation
[39]	Classification: 3D ResNet-18	Classification accuracy: 0.9924, recall: 0.9996, precision: 0.9935, F1-sorce: 0.9965	Model is still a black box
Proposed	Classification: ORB + BOF + PNN, Infection extraction: Semantic segmentation using DeepLabv3+ with weights initialized by ResNet-50	Classification accuracy: 0.997, AUC: 0.9988, sensitivity:0.999, specificity: 0.996, PPV:0.996, NPV: 0.999. Infection extraction: global accuracy:0.9947, weighted IoU: 0.9899, mean BF score: 0.9453	Imbalanced dataset of different stages of COVID-19 for infection extraction model

The pixel-labelled datasets of COVID-19-infected regions CT images from different disease stages was imbalanced. Due to the pandemic, few radiologists were available to obtain ground truths of more data. Once this is achieved, research in the area of infection extraction can be developed by applying new techniques so that severity identification issues faced by hospitals can be solved effectively. Future research should look at developing an efficient DL CNN which can perform classification along with more accurate infection extraction. We could also consider the averaging of different DL models for this application.

## Conclusions

We have proposed a fully automatic CAD system which is able to perform COVID-19 diagnosis from lung CT images, along with infected region extraction, severity score prediction and disease stage identification. The model was developed from a publicly available dataset which includes data from different cohorts; hence, it achieves diversity, and our developed model is more accurate, applicable to real-time applications. Since the ML classifiers were trained using the feature from SIFT, SURF, and ORB, the model remains invariant to the scale, rotation, noises, etc. of dataset images. So far, this work is the first framework utilizing image descriptors and bag of features technique for COVID-19 diagnosis application. The model achieved a classification accuracy of 99.7%, with a misclassification rate of 0.0027. Once the patient is diagnosed as positive, this model automatically extracts the COVID-19-infected region and identifies the disease stage as mild, moderate, or severe. Our infection extraction model achieved the following segmentation performance measures: a weighted IoU value of 0.9899 and a mean BF score of 0.9453. We combined conventional ML and DL in this study to obtain the best complete model. As a part of the CAD system development, also developed a DL semantic segmentation model which is able to perform automatic lung segmentation from a CT image. This model is applicable for all AI applications related to image pre-processing of lungs in the fields of biomedical image processing. This model could be employed in hospitals to automatically detect COVID-19 cases and identify the disease stage. Moreover, patients will be given appropriate treatment, based on the severity level, without any delay.

## Data Availability

The full CT image dataset used in the proposed study are publicly available at the China National Center for Bioinformation at http://ncov-ai.big.ac.cn [27].

## Abbreviations

CAD: Computer-aided diagnosis
COVID-19: The coronavirus disease of 2019
GGO: Ground-glass opacities
CL: Consolidation
ML: Machine learning
CT: Computed tomography
AUC: Area under the curve
IoU: Intersection over union
BF: Boundary F1
SIFT: Scale-invariant feature transform
SURF: Speeded-up robust features
ORB: Oriented FAST and rotated BRIEF
CNN: Convolutional neural network
RT-PCR: Reverse transcription-polymerase chain reaction test
SARS-CoV-2: Severe acute respiratory syndrome coronavirus 2
ACE2: Angiotensin-converting enzyme 2
AI: Artificial intelligence
CXR: Chest X-ray
ROI: Region of interest
DL: Deep learning
FCN: Fully convolutional networks
ASPP: Atrous spatial pyramid pooling
GS: Grid search
